# At the Heart of It All: Emotions of Consequence for the Conceptualization of Caregiver-Reported Outcomes in the Context of Colorectal Cancer

**DOI:** 10.3390/curroncol28050355

**Published:** 2021-10-16

**Authors:** A. Fuchsia Howard, Kelsey Lynch, Scott Beck, Maria-José Torrejón, Jonathan Avery, Sally Thorne, Antony Porcino, Mary De Vera, Leah Lambert, Angela Wolff, Melanie McDonald, Joyce Lee, Penelope Hedges, Michael McKenzie

**Affiliations:** 1 School of Nursing, The University of British Columbia, Vancouver, BC V6T 2B5, Canada; Kelsey.Lynch@ubc.ca (K.L.); Scott.Beck@bccancer.bc.ca (S.B.); M.torrejon@alumni.ubc.ca (M.-J.T.); Jonathan.Avery@ubc.ca (J.A.); Sally.Thorne@ubc.ca (S.T.); Leah.lambert@bccancer.bc.ca (L.L.); Angela.Wolff@twu.ca (A.W.); 2 BC Cancer, Vancouver, BC V5Z 4C2, Canada; Antony.Porcino@bccancer.bc.ca (A.P.); Melanie.McDonald@bccancer.bc.ca (M.M.); JLee2@bccancer.bc.ca (J.L.); penelope@idtcanada.com (P.H.); Mmckenzi@bccancer.bc.ca (M.M.); 3 Department of Supportive Care, Princess Margaret Cancer Centre, University Health Network, Toronto, ON M5G 2C1, Canada; 4 Faculty of Pharmaceutical Sciences, The University of British Columbia, Vancouver, BC V6T 1Z3, Canada; Mary.Devera@ubc.ca

**Keywords:** supportive care needs, patient, family, caregiver, qualitative research, emotions, caregiver reported outcomes, colorectal cancer, oncology, psychosocial

## Abstract

Colorectal cancer (CRC) can be demanding for primary caregivers; yet, there is insufficient evidence describing the caregiver-reported outcomes (CROs) that matter most to caregivers. CROs refer to caregivers’ assessments of their own health status as a result of supporting a patient. The study purpose was to describe the emotions that were most impactful to caregivers of patients with CRC, and how the importance caregivers attribute to these emotions changed from diagnosis throughout treatment. Guided by qualitative Interpretive Description, we analyzed 25 caregiver and 37 CRC patient interviews, either as individuals or as caregiver-patient dyads (six interviews), using inductive coding and constant comparative techniques. We found that the emotional aspect of caring for a patient with CRC was at the heart of caregiving. Caregiver experiences that engendered emotions of consequence included: (1) facing the patient’s life-changing diagnosis and an uncertain future, (2) needing to be with the patient throughout the never-ending nightmare of treatment, (3) bearing witness to patient suffering, (4) being worn down by unrelenting caregiver responsibilities, (5) navigating their relationship, and (6) enduring unwanted change. The broad range of emotions important to caregivers contributes to comprehensive foundational evidence for future conceptualization and the use of CROs.

## 1. Introduction

Growing evidence suggests that using patient-reported outcomes (PROs) in oncology can lead to significant and tangible improvements in patient outcomes, care satisfaction, quality of life, and even survival [1,2,3,4,5,6]. PROs are assessments of health status or health-related quality of life from the patient’s perspective [7]. While the concept of patient-and-family-centered care is increasingly supported [8], the large majority of PRO research in oncology focuses on the patients themselves. The incorporation of primary caregiver outcome measures, termed caregiver-reported outcomes (CROs), has received little attention [9]. CROs specifically refer to caregivers’ assessments of their own health status and health-related quality of life as a result of supporting a patient with a serious illness. We use the term primary caregiver to refer to key family members or friends who play important roles in the care team, including providing ongoing emotional and functional support throughout the cancer trajectory [10].

Primary caregivers, hereafter referred to as a caregiver, provide about 70% to 80% of patients’ cancer care, taking on a vast array of responsibilities and complex illness management roles, yet most caregivers receive minimal formal training [11]. Caregiving responsibilities often include collecting and managing information, driving, and accompanying the patient to medical appointments, providing personal and medical care in the home, and providing emotional and spiritual support. Despite their best efforts, caregiving demands can inadvertently result in high caregiver burden, defined as the extent to which caregivers perceive that their emotional, physical, social, financial, or spiritual functioning is adversely affected as a result of caregiving [12]. The combined effect of these difficulties may lead to further physical and psychological decline in the caregiver, with some research suggesting that higher burden is associated with increased caregiver morbidity and mortality risk [13,14]. The stresses from caring for someone with cancer can negatively affect psychological well-being, with emotional distress most commonly assessed via caregiver anxiety and depression [13,15,16,17,18]. In their review, Girgis and colleagues [17] reported the prevalence of anxiety and depression among caregivers of cancer patients as ranging from 10% to 53%, noting that in some studies, rates of anxiety and depression exceeded that of patients. In addition to the focus on emotional distress, other difficult emotions documented include worry, fear, feelings of being overwhelmed, uncertainty, and hopelessness [18]. However, it is not fully understood how the importance of the various emotions experienced by caregivers change at different times across the cancer trajectory [19]. 

A recent international Delphi study of priorities for cancer caregiver research identified the need for research on routine screening with CROs to identify caregivers at greatest risk of burden [20]. Yet, there is a remarkable lack of evidence about what outcomes matter most to caregivers, and how to integrate CROs into cancer care workflow and processes. It is conceivable that measuring CROs to help guide care interactions, decisions and interventions in cancer care could be associated with significant benefits for patients, caregivers, and health service outcomes. Our team launched a study with the overarching objective of describing the outcomes most important to caregivers, and how to integrate CRO measurement across the care trajectory to increase the probability of meeting primary caregivers’ needs. We focused specifically on caregivers of patients with colorectal cancer (CRC) because they play considerable and changing roles from the time of diagnosis through treatments, well into survivorship or near end-of-life. In Canada, CRC is the third most commonly diagnosed cancer [21]. Initial treatments may be complex, and surgery is almost always performed, which may necessitate an ostomy. In more extensive disease, surgery is often combined with chemotherapy and radiation therapy. Subsequent metastatic disease is common; despite multiple possible lines of palliative chemotherapy, symptom burden is high, and survival is usually not more than a few years [22]. CRC is a disease for which caregiving can be demanding and is associated with high caregiver burden; however, there is insufficient caregiver-perspective evidence describing the trajectory of burden, acceptable means of assessment, and desired support. 

In this study, we found that the emotional impact of being a caregiver to a patient with CRC stood out as central to caregiver experiences. Thus, the purpose of the analysis we report on here is to describe: (1) the emotions that appeared most impactful to caregivers of CRC patients, and (2) how the importance caregivers attribute to these emotional outcomes changed over the cancer trajectory. We do not describe the caregiver experiences of when the patient is in the survivorship phase or near end-of-life as part of this analysis because the post-treatment phases are distinct with differing emotional impacts on caregivers. These will be described in future publications. Of note, though we recognize that the conceptualization of emotion remains contested, we consider emotion as a complex reaction pattern elicited from an experience or an event, with an emotional stimulus triggering behavioral, physiological, and subjective (e.g., thoughts) responses [23,24].

## 2. Materials and Methods

### 2.1. Design

This qualitative, Interpretive Description [25] study was guided by the principles of patient-oriented research [26] such that we engaged researchers, caregiver- and patient-partners, and multidisciplinary stakeholders as equal team members throughout the research lifecycle, intending to focus on priorities of our caregiver- and patient-partners and to generate knowledge that will be applied to improve caregiver care and outcomes. We chose Interpretive Description because it is an applied qualitative approach to constructing experiential evidence relevant to practice disciplines and clinical application [25]. Interpretive Description allows for interpretation and explanation that rests on the epistemological directionality of the applied disciplines, rather than an extant theoretical framework [25]. Of note, the majority of the research was conducted virtually because of restrictions due to the COVID-19 pandemic. 

### 2.2. Setting and Sample Recruitment

This research was conducted in British Columbia, Canada, wherein there is a public health care system that provides universal health care inclusive of oncology care through a province-wide cancer organization that serves a population of roughly 5 million. The study was reviewed and approved by the harmonized University of British Columbia, BC Cancer institutional ethics review board. We recruited caregivers who were involved in providing care to a patient with CRC (e.g., spouse, parent, sibling, adult child, unmarried partner, neighbor, or close friend). We included patients who were diagnosed with CRC and ≥19 years of age. Further inclusion criteria for both caregivers and patients were the capacity to give informed consent and speak English. For feasibility reasons, we recruited caregivers and patients who had completed primary CRC treatment and asked them to reflect upon the key time points across the CRC trajectory, rather than recruiting participants at different time points which would require a much larger sample. While we purposefully intended to recruit through various oncology and community healthcare settings that represented diversity in participant characteristics and experiences, owing to the COVID-19-related restrictions, we pivoted to recruiting entirely online and were reliant on a more convenience approach to sampling. This included study advertising in online newsletters and social media pages of provincial and national caregiver and oncology organizations as well as emailing the study poster to a listserv of individuals who previously consented to being contacted for future research purposes. 

### 2.3. Data Collection

We conducted in-depth, semi-structured interviews with caregivers and patients virtually via Zoom (video-conferencing platform, audio only) from April 2020 to September 2020. We co-created interview guides drawing from our clinical and research expertise as well as input from our caregiver- and patient-partners and healthcare providers. Examples of the caregiver questions included: (1) Tell me about the main challenges you struggled with as a caregiver when your loved one was first diagnosed. (2) How were those challenges different from the challenges you faced during treatment? (3) What about now (after treatment/near end-of-life)? (4) How did the importance of those challenges change? (5) What has helped you cope with the challenges you have faced? (6) What factors (e.g., challenges and positive aspects of caregiving) would you like healthcare providers to ask you about? (7) When would this be helpful/unhelpful to you? (8) What would you like healthcare providers to do with this information? (9) What type and when would support be helpful/unhelpful to you? We asked patients similar questions but reflected on what they perceived to be caregivers’ experiences and needs. We modified our interview guides based on preliminary insights identified during ongoing analysis. This included asking interview questions in subsequent interviews that sought further exploration or comparison to the initial inductive interpretations. Interviews lasted 45 to 90 minutes and were recorded, transcribed verbatim, de-identified, and checked for accuracy. For caregivers who volunteered along with their patient, we offered them the option of individual or dyad interviews (that is, together in the same interview), intending for half of the interviews to be conducted with caregiver-patient dyads. Overall, the interview data collected were high in information power [27] owing to the study participants’ first-hand experiences and highly detailed accounts relevant to the research aims, the knowledge and skills of the interviewers, the highly detailed accounts of the study participants, and the variation in experiences represented in participants’ accounts. 

### 2.4. Data Analysis

A group of five team members involved in more in-depth analysis identified, discussed, and developed an initial coding frame that included the very broad descriptive themes initially evident in the data, with the emotional aspect of caregiving being one. This initial descriptive coding frame was also revised based on caregiver- and patient-partner perspectives from reading a subset of transcripts. Using a stratified analysis approach, we began the more in-depth analysis of this broad theme on an initial set of 20 transcripts (from interviews with 22 participants, 12 of which were caregivers and 10 were patients) by identifying transcript segments that reflected emergent patterns, diversities, and examples. This informed our development of a coding frame that was then applied to the 20 transcripts using the process of constant comparison, comparing and contrasting pieces of data, within and across participants. This iterative process resulted in the grouping and regrouping of analytic codes into categories, which we then applied to a subsequent 18 transcripts (from interviews with 22 participants, 13 of which were caregivers and 9 were patients). We applied the categories to the remaining 18 patient transcripts but did not analyze this data until we were refining our analysis. The ongoing comparing and contrasting of participants’ experiences and the ideas within the categories were further facilitated by intentionally aiming for a higher level of conceptualization and interpretation, that is, moving from descriptive analysis to interpretive analysis [25]. During the process of writing our findings in manuscript form, we reflected on our interpretations with data from the remaining 18 transcripts and refined the findings into an interpretive description of the important emotions for caregivers of patients with CRC. In our stratified approach to analysis, we intentionally analyzed interview data from both caregivers and patients during each step. That is, the initial analysis was based on earlier interviews with 12 caregivers and 10 patients, the second round on successive interviews with 13 caregivers and 9 patients, and the final refining of analysis on 18 patients. The number of transcripts (56) does not match the total number of participants (62) because 12 participants chose a dyad interview. 

Throughout the analysis process, the five-member in-depth analysis group discussed the analysis and emergent findings during bi-weekly group meetings. Our evolving analysis was also brought back to the full team for input, including the caregiver- and patient-partners and multidisciplinary healthcare providers, who complemented and challenged our insights and emerging findings. Additional meetings with two researchers and our caregiver- and patient-partners were conducted specifically to facilitate meaningful input from caregiver- and patient-partners. All team meetings were typified by open dialogue and discussion, which facilitated an interpretive analysis wherein we engaged with data in collaboration with the multiple perspectives and professional/lived experience expertise of our multidisciplinary team and partner members.

## 3. Results

### 3.1. Sample Demographic Description 

A total of 62 individuals (25 caregivers and 37 patients with CRC) participated in this research (see Table 1). Only 12 of the 62 participants chose a caregiver-patient dyad interview. The mean age of caregivers was 55 years (range 27–79 years), and the majority identified as being a woman (*n* = 22), a caregiver to one’s partner (*n* = 17), married or common-law (*n* = 20), in the same home as the person being cared for (*n* = 16), and in a large urban city (*n* = 14). The mean age of CRC patient participants was 65 years (range 31–84 years), and the majority identified as being a man (*n* = 21), the partner to the caregiver (*n* = 26), married or common-law (*n* = 27), in the same home as the caregiver (*n* = 29), and in a large urban city (*n* = 20). Among patient and caregiver participants, there was a range of cancer stages, and 29 had a colostomy or ileostomy at some point.

From the interviews, it was clear that the emotional aspect of caring for a patient with CRC was central to, and at the heart of, the caregiver experience. We present our interpretation of caregiver experiences that engendered emotions of consequence, that is, emotions and the feelings participants expressed that were central and also of value when considering CROs. We describe the caregiver experiences that engendered emotions of consequence at the time of diagnosis; (1) facing the patient’s life-changing diagnosis and an uncertain future; then, during CRC treatment, (2) needing to be with the patient throughout the never-ending nightmare of treatment, (3) bearing witness to patient suffering, and (4) being worn down by unrelenting caregiver responsibilities. We then describe caregiver experiences of (5) navigating their relationship, and (6) enduring unwanted change that engendered emotions of consequence, throughout the CRC trajectory. Throughout the description of these six themes, we highlight key contributing factors and/or circumstances, though of note, some of these factors and/or circumstances were evident across themes. See Table 2 for an overview of findings.

### 3.2. Caregiver Experiences That Engendered Emotions of Consequence during CRC Diagnosis and Treatment 

#### 3.2.1. Facing the Patient’s Life-Changing Diagnosis and an Uncertain Future

The time of CRC diagnosis was unsurprisingly a time of emotional turmoil for caregivers wherein life going forward took a striking, unexpected turn. For many caregivers, particularly those who were unfamiliar with cancer or who had minimal experiences of cancer in their family, the unimagined diagnosis “came out of the blue” and/or engendered shock, panic, fear, distress, and anxiety, and it was difficult to “accept” and make sense of. Almost all caregivers were stunned by the diagnosis in instances where the patient had kept hidden their “embarrassing and private” symptoms, leaving some feeling unprepared to step into the caregiver role. Moreover, caregivers felt forced to imagine a future without their loved one and altered family arrangements, with the threat of death particularly distressing in situations where the patient was a primary caregiver for other family members. For example, a spousal caregiver feared losing their wife, but also feared their children losing their mother. The diagnostic and treatment planning process took time and several procedures for some, and the longer the wait, the more unsettling for caregivers.

Caregiver uncertainty when waiting for a CRC treatment plan contributed to feelings of powerlessness and helplessness because, as one 67-year-old woman spousal caregiver stated, “*there’s nothing we can individually do about it because that’s totally beyond our control*” (Participant 15). Not knowing what to expect in terms of when treatment would begin, what it would entail, and how it would affect the patient provoked caregiver fear. Recognizing the need to re-orient their lives around the patient’s CRC treatment, yet being unable to envision the path forward, left most caregivers uncertain and worried about how they and other family members would manage. These emotions were further exacerbated when treatment included the possibility of an ostomy owing to unfamiliarity with what an ostomy was, ostomy care and management, and possible ostomy-related life changes. While information pertaining to the patient’s cancer treatment was readily available and greatly valued by many, many participants contrasted this with the lack of caregiver-specific information that would have helped them prepare to be a caregiver and familiarize themselves with support or resources. Overall, the patient’s life-changing CRC diagnosis gave rise to a multiplicity of complex emotions including shock, panic, fear, anxiety, distress, worry, uncertainty, and powerlessness.

#### 3.2.2. Needing to Be with the Patient throughout the “Never-Ending Nightmare” of Treatment

For most participants, caregiver emotional upheaval eased somewhat as they became accustomed to new routines that revolved around cancer treatments, and their worry and distress was more episodic when treatment complications arose. Despite these accounts, many caregivers, including those who described their acute worries and anxieties easing somewhat, also continued to fear their loved one dying. Caregiver worry and distress manifested as repetitive negative thoughts, sleeplessness, constant checking and monitoring of the patient, and the feeling of needing to be with the patient at all times. Caregiver hypervigilance over weeks and even months slowed down their sense of time such that the period of treatment was perceived as stretched, lasting a long time and constituting “a never-ending nightmare”. 

Despite recognizing that ever-presence was not always realistic, most caregivers re-oriented their lives and made concerted efforts to stay by the patient’s side throughout all aspects of treatment, when they were at home as well as during any other outings or activities. One 65-year-old patient recounted his wife’s efforts to be ever present throughout treatment, including the time of surgery:

*We’ve been together over 40 years. [Emotional]. She’s (wife/caregiver) been beside me every second. After my diagnosis she was very supportive. “[Participant’s Name] we’ll get through this.” When I was in the hospital, she was there day and night [Crying]. For surgery, of course she took me to the hospital and my surgery was supposed to be four hours. It ended up just over eight hours. So she sat in the waiting room that whole time. And when I woke up, she was there right beside me. And then every day she was there. [Crying].*

(Participant 43)

Caregivers reasoned that because they knew the patient best, their presence was vital to “keep an eye” on the patient to monitor for side effects, ensure they were tolerating treatment, prevent the patient from injury, and offer support and reassurance. Worry and anxiety intensified when the caregiver did not live with the patient; the caregiver was unable to accompany the patient because of other family or employment commitments; or the patient had to travel for treatment. For example, one 64-year-old woman caregiver described the distress of getting time off work to accompany her husband throughout treatment:

*I ended up using my sick days and I went there and stayed there and I took time off work, but that was the only way I could do it in the job I’m in... It did work out in the long run [eventually lost employment], but it was stressful… I was going to be so anxious and feel so much anxiety, not being able to go, that the doctor gave me a letter saying that I would be… stressed out. I would be getting time off because there was no way I could be working and be worrying about him in the hospital.*

(Participant 57)

As evident in this quote, caregiver concern about the patient could be all-consuming, crowding out other responsibilities. There was also a loss of reciprocal emotional support between some caregivers and patients, such that a changed relationship dynamic and physical separation prevented the caregiver from feeling supported by the patient. For many caregivers, being with the patient throughout treatment tempered the psychological burden. Yet, a few participants described the emotional toll wherein caregiver mental health declined as the patient progressed through treatment. These caregivers included individuals with a pre-existing mental health condition as well as those who had a previous traumatic cancer experience, such as the death of a loved one when they were young.

#### 3.2.3. Bearing Witness to Patient Suffering

The study participants conveyed the emotional pain caregivers experienced when bearing witness to the physical, cognitive, emotional, and existential suffering of the patient throughout CRC treatments. This was particularly distressing in instances wherein caregivers came to see the patient as they never had before—ill and unable to function as usual, as depicted by a 65-year-old woman caregiver for her husband, *“he has such a strong will to do everything that—just seeing him so weak and so tired—that was harder on me than anything”* (Participant 28). Caregiver suffering, sadness, and frustration were highlighted when the patient experienced significant treatment side effects and invasive medical interventions, and when patient suffering was considered unnecessary. As a 27-year-old one woman caregiver for her mother recounted:

*I’m sitting there watching my mother who, over probably the last year, she’s probably lost at least 20 pounds from in and out of the hospital, from sepsis, from liver failure, from all of these things, from being NPO-ed [nothing by mouth] for 24 hours. She doesn’t have a lot of extra to spare. It’s gotten more and more frustrating as we sit there and we wait and if she can’t eat for a procedure for a while, that’s fine. But then when you cancelled the procedure, that’s really, really frustrating. And it’s really hard emotionally for everyone. Because if I have to watch my mother suffer, it better be for a reason, right? It better be because we’re doing a procedure to make her feel better, to have her quality of life be better, not we’re doing it as an experiment.*

(Participant 60)

Moreover, caregiver distress arose for some in circumstances wherein patients’ functioning significantly declined, such that they were no longer independent, and the caregiver took on a new role of looking after the patient. This sudden and unexpected role reversal was disorienting, as described by a 40-year-old daughter who cared for her mother:

*It can be very distressing. Seeing my mom physically decline was very upsetting because she was very strong. It was a very weird position to be in because it felt like a role reversal or suddenly, very suddenly, went from being the kid, even though I was like 35, to feeling more like a parent. It kind of messes with your head because it’s not how things have always been and it’s a quick adjustment period and you’re just sort of thrown into the situation. She also had bleeding, like rectal bleeding and that is pretty upsetting to see. It was upsetting for her. She went into wearing Depends® type of underwear and that was upsetting for her and for me.*

(Participant 4)

In other instances, caregivers described not only bearing witness to the suffering of the patient, but also experiencing or taking on the patient suffering themselves. Patient and caregiver experiences were inextricable linked, and the separation between whose emotions were whose became blurred. For example, a 58-year-old man who was the caregiver for his wife commented that the emotions of his wife, “*would manifest itself in both of us, ‘cause at some point in time, whatever’s wrong with her would be wrong with me, somehow. That it may either be angry or upset or quiet or whatever*” (Participant 31). The emotional pain of bearing witness to patient suffering seemed to stay with caregivers, some of whom recounted vivid, detailed, often visual memories of the time of treatment months or even years later. 

#### 3.2.4. Being Worn Down by Unrelenting Caregiver Responsibilities 

A subset of participants highlighted caregivers feeling overwhelmed and exhausted as treatment progressed and when medical issues or complications arose and persisted. The ongoing and unrelenting nature of caregiving was both emotionally and physically demanding, and the longer the demands continued, the more difficult it became for caregivers who became exhausted and “worn down” by “day in and day out” caregiving. As one 33-year-old woman caregiver for her husband recalled,

*He [patient] was on a lot of opiates and he was having panic attacks that they didn’t really know how to control. So when he would wake up for panic attack, he’d wake me up and we’d sit down and we’d talk and we’d watch a movie and we’d calm down, and try to sleep again, but I wouldn’t really sleep cause I was stressed. And then I’d be up early. And so I guess I didn’t sleep very much for those two years.*

(Participant 50)

The above quote brings into focus the stress of perceiving oneself as the “sole” caregiver and the enormity of the caregiver role. While some welcomed being the primary and sole caregiver, for others, feeling “responsible for everything” left the caregiver also feeling alone, unsupported, and depressed, and on the verge of emotional and physical collapse. Further exacerbating feeling overwhelmed and exhausted was the lack of recognition and appreciation from other family members as well as healthcare providers. Thus, caregivers felt invisible and expendable, which eroded their sense of self-worth as a carer but also as someone worthy of care and support themselves. 

Feelings of being overwhelmed and exhausted accumulated as caregivers juggled multiple competing demands, such as employment and caring for other family members, and they did not have the time or energy to be ever-present. Caregivers frequently expressed being unable to seek support because they were too busy and overwhelmed, and all of their thoughts and energy were taken up by caregiving. One 37-year-old woman caregiver to her husband highlighted that:

*If you don’t have anyone to help you with the sick person and the kids, it feels a little bit overwhelming to be told, “well, make sure you’re taking time for yourself, eat well, exercise every day, take care of your husband, take care of your kids, try to not lose your job, make sure you pay all the bills, get enough sleep, go to yoga. Oh. And also take some time for yourself.” And it’s like, there aren’t that many hours in the day. So I would feel like, okay, thank you for the advice, but that’s not necessarily feasible.*

(Participant 59)

Other participants similarly highlighted that the commonly endorsed notion of individual responsibility wherein caregivers were encouraged to look after themselves and to engage in self-care was not only unhelpful but completely unrealistic. Yet, one of the most common recommendations participants gave in interviews was for caregivers to do just that, highlighting the pervasive focus on self-management as an acceptable and endorsed solution. Further, some caregivers expressed their reluctance to discuss their thoughts and feelings with the patient or other family members out of fear of burdening them, resulting in some downplaying and/or concealing their emotions and contributing to the caregivers’ sense of loneliness. Despite multiple competing demands and the enormity of caregiving, the minimal opportunities for reprieve and emotional support for caregivers exacerbated the emotional and physical exhaustion.

### 3.3. Caregiver Experiences That Engendered Emotions of Consequence Throughout the CRC Trajectory 

We identified emotions of consequence that appeared related to aspects of caregiving, namely navigating their relationship with the patient and enduring unwanted change, more so than the specifics of CRC diagnosis or treatment. In navigating their relationship with the patient, caregivers commonly experienced tension and conflict with others, anger and frustration, and guilt, while loss and grief were prominent as caregivers endured unwanted change. 

#### 3.3.1. Navigating Their Relationship 

Many of the study participants either explicitly highlighted or alluded to interpersonal tension and conflict between the caregiver and the patient throughout the CRC trajectory. This tension and conflict often arose with changes in the roles and responsibilities of both the caregivers and patients, particularly when what the caregiving offered did not match the patient desire for either support or independence. Caregivers described their awareness of the patient’s vulnerability and need for advice and/or care, which was at times at odds with the patient’s desire for independence and autonomy. The mismatch between the caregiver’s perception of the patient’s need for care versus the patient’s desire for independence became especially apparent when caregiving involved physical care of private body parts or was related to bowel functioning or ostomy management. These aspects were described as embarrassing, humiliating, and degrading for the patient, as recalled by a 67-year-old woman caregiver for her partner who identified as a woman:

*She [partner] is not a good patient. She doesn’t want to be a patient, so I don’t want her to be a patient, but sometimes I’ll say something like, “well, why didn’t you take Imodium? Because you know you really need some now.” And it’s like, “no, I took it once and it helped, but I didn’t like what it did and I’m not taking it again”… She’s very stubborn. So part of it is for me to figure out how much to interfere or, if she’s feeling horrible because she’s running to the toilet all day and I know that an Imodium would help and I’ve said it once or twice and she’s not interested, then I just have to give it up and go for a walk because it makes me kind of frustrated… For me, the frustration comes when I know that she’s not doing something that would help.*

(Participant 54)

A 53-year-old non-binary caregiver for their partner further explained this:

*He’s a little hard to help because he’s very independent. I think he doesn’t, like most of us, he doesn’t like being vulnerable. This has also been a source of conflict in our relationship... He likes to do things himself. He’s a very private person, especially about his body. And the fact that this is rectal is just, for him, horrible, especially humiliating.*

(Participant 47)

Several caregivers described feelings of helplessness, “because there’s nothing you can do. You’re not healing. You’re not going through it. You’re just sitting and watching and that’s in a way, that’s kind of harder” (Participant 15). Overall, the interviews suggest that caregiver feelings of helplessness or usefulness were influenced by either the patient’s resistance to or acceptance of caregiver support. Further, feelings of helplessness were apparent when notions of care were primarily related to medical care that was considered in the domain of the healthcare team, while feelings of usefulness were apparent when notions of care were, for example, more inclusive of companionship and emotional support, the management of information, and pragmatic support (i.e., driving and accompanying the patient to appointments). 

Interpersonal tension and conflict were further depicted as arising when there was a mismatch in how the caregiver and the patient approached and coped with the difficulties associated with cancer. These were expressed as differences in preferences, such as talking about emotions versus not, focusing on the best possible outcome versus preparing for the worst, considering the most likely outcomes versus planning for all possibilities, and being positive versus being realistic. To mitigate this tension, some caregivers described their concerted efforts to change their approach to providing support, prioritizing the patient’s needs over their own, so that it aligned with what the patient indicated was most helpful. This was highlighted by a 53-year-old non-binary caregiver for their husband:

*It’s hard to learn to just shut up and just nod your head and put your hand out on their knee and hug them and not say anything. It’s hard to resist the urge to say reassuring things, but he was able to be very clear with me that I need you not to do that… Initially it upset me because I felt like, you don’t recognize that I’m loving you. I might have initially had that reaction, but I’m at a place in my life where I recognize that that’s just my reaction and I just need to hear what he has to say. It’s not my feelings that matter right now. It’s his, and that’s fine.*

(Participant 47)

In contrast, other caregivers who recognized the incongruence of their coping strategies with that of the patient described their efforts to work through their emotions separately from the patient so that both could employ strategies they found helpful but also to avoid conflict. One 27-year-old woman caregiver described how necessary it was for her and her mother to acknowledge and cope with the emotional aspects of cancer at the same time, but separately, through processes that “*couldn’t intersect*” (Participant 60).

While several caregivers spoke freely about their anger and frustration with the patient at various times throughout the CRC trajectory, others were somewhat tentative and self-conscious divulging these emotions in the interviews. Anger was conveyed as an unwelcome and “uncomfortable” emotion that was framed as an unproductive expression, a hindrance to caregivers, and an unacceptable emotion because of fear of doing anything that could upset or compromise the patient’s well-being. Commonly, there appeared a hierarchy of roles wherein the patient role trumped the patient-relationship role (i.e., as spouse, child, or parent) such that caregivers were expected to uphold and attend to their loved one as a patient first and foremost. The primacy of the patient role was reflected in the comments of a 60-year-old woman who was the caregiver for her husband:

*There can be genuine anger with your partner. There’s that sense that because he’s sick, you have to be sweet and kind. You can’t express any unpleasant emotions because that will be damaging to his healing so that that adds another layer to it… He really struggles with, “don’t be, don’t be angry with me. Oh, how can you be basically be unkind. I’m the patient here,” which yes. I know. I get that right. But, we still have a relationship and in relationships, sometimes you have disagreements. That makes it difficult to navigate.*

(Participant 5)

In response to their own anger, some caregivers detailed their conscious efforts to diffuse their anger, usually by focusing on acceptance, so that it would not interfere with the care they were providing. 

Yet, the lack of available professional support for caregivers in this regard was notable, particularly considering that some caregivers felt anger was not something that could be discussed openly with the patient, other family members, or even close friends. 

Study participant accounts revealed caregivers’ feelings of guilt, though caregivers’ explicit expressions of guilt were often avoided during interviews. Caregiver guilt stemmed from recognition of how ill the patient was while they themselves remained healthy and functioning, particularly in instances wherein the caregiver was older, was a parent, or perceived themselves as less healthy compared to the patient prior to CRC. Moreover, caregiver guilt was highlighted among caregivers who were struggling emotionally but who were also of the opinion that as the caregiver they had to be “strong” and composed to support the patient. These caregivers expressed feelings of guilt when they were unable to minimize their strong emotions, fearing that they would burden the patient with their own suffering, particularly when they thought the patient was “going through so much worse”. Lastly, although there was overwhelming acknowledgment of the necessity for caregivers to attend to responsibilities not related to caregiving, take time away from caregiving, and engage in self-care activities, these were reportedly associated with caregiver guilt, as described by a 60-year-old woman caregiver for her husband:

*Of course you would give up everything. Why wouldn’t you do that? This is a person that you love. So, there is a degree of guilt I think because, I’m not going to sacrifice absolutely everything I’ve fought so hard over the years to build up. So, there is a sense of guilt to a degree and this will take everything. I still feel guilty because I feel like I should be sitting there holding his hand whenever I have a spare minute, but I just can’t, it’s not realistic. So, there is lots of guilt that goes with this whole, so journey to use the metaphor. So anger and guilt.*

(Participant 5)

Some caregivers described their guilt when they were unable to fulfill responsibilities related to work or their other family members, such as spending time with young children or managing the household, in order to carry out caregiving responsibilities for the patient or when they simply could not be with the patient at all times. The feelings of guilt were unintentionally reinforced by family or even the patient when, for example, they questioned where the caregiver had been or what they had been doing when they were not with them.

#### 3.3.2. Enduring Unwanted Change 

Cancer was a marked and ongoing life disruption that forced caregivers to question their own hopes and dreams involving the patient, including sharing a life, creating a home, raising a family, retiring, growing old, and traveling together. Throughout the CRC journey, it became clear for some, particularly in situations where the patient’s cancer was no longer curable, that the future they had envisioned was untenable. Most caregivers described how they shifted their priorities and reoriented their life to care for their loved one, which resulted in new daily and weekly routines throughout treatment. Caregiver feelings of loss and grief were described in situations wherein caregivers put their “*life on hold*”, stopped or cut back on their work, or were no longer able to participate in meaningful activities that enhanced their sense of self. One 47-year-old woman reflected on her loss of self-identity as she assumed the caregiving role for her mother:

*I love the work that I do. I love the youth. And so having to step away from that and step into the caregiving piece to not have that aspect of my identity any longer was really challenging…. It was just challenging to not have that piece of who I am and to feel fulfilled in that way, to feel validated. I didn’t realize how much of that was my identity until I suddenly stepped away from it.*

(Participant 64)

Further feelings of loss and grief were expressed when caregivers considered their life and lifestyle forever changed. Forced changes in lifestyle and the inability to engage in usual or meaningful activities were most pronounced in situations wherein unpredictable bowel functioning made leaving the home almost impossible. This was often the case when the patient and caregiver experienced problems learning about and managing the patient’s ostomy, as reflected in the accounts of a 64-year-old woman caregiver for her husband:

*With the colostomy, that poses a lot of problems for doing things. It’s a big life changer for him. And for going places, you always have to be prepared. It’s not a very easy one. It’s not on a schedule and he has had a lot of trouble cause it herniated, so clothes don’t fit. Probably that’s been a big hassle. He’s done pretty good, but you can’t do the same things you used to do. Let’s say swimming and stuff, forget it, that’s not gonna happen. Or, if he does too much exercise or something, then the colostomy it leaks or whatever… that’s why I had to stop working. It was so unpredictable.*

(Participant 57)

Fewer opportunities for the caregiver to leave the house or participate in activities meant fewer occasions to engage with other family and friends, contributing to feelings of loss of friendships and social disconnectedness. With so much time and energy dedicated to caregiving, there was also limited time left for caregivers to connect with their social network. Moreover, CRC treatment and care were sometimes depicted as a private matter, the details of which could not easily be shared with others, particularly those who had never had a similar experience. Thus, some caregivers felt alone and unable to share their emotions with someone with whom they could relate even if there were people around.

Finally, among caregivers of their spouse or partner, a subset described further loss and grief stemming from the putting on hold of physical intimacy with their partner. For these caregivers, the pause in physical intimacy began prior to the CRC diagnosis, when the patient had symptoms of pain, fatigue, and bowel irregularity, and continued throughout treatment and especially when the patient had an ostomy or experienced a progressive health decline. While some grieved the permanent shift in physical intimacy, others attended to physical intimacy to maintain hope, as depicted by a 67-year-old patient who identified as a man:

*I think she initiated sex fairly quickly after I got home. She initiated it in terms of just wanting to return to normal. I thought that was maybe a clue in terms of her perspective as just wanting to resume, to continue for me to be healthy.*

(Participant 39)

## 4. Discussion

These findings contribute to a comprehensive understanding of the emotional aspects of caregiving that are considered important by caregivers themselves, as foundational knowledge for future conceptualization of CROs. In summary, from both caregiver and patient perspectives, emotional aspects are central and at the heart of caregiving for a person with CRC. There were caregiver experiences that engendered emotions of consequence during CRC diagnosis and treatment, specifically, (1) facing the patient’s life-changing diagnosis and an uncertain future, (2) needing to be with the patient throughout the never-ending nightmare of treatment, (3) bearing witness to patient suffering, and (4) being worn down by unrelenting caregiver responsibilities. Of note, CRC treatment was not conceptualized as it usually is by health care providers—diagnosis, surgery, chemotherapy, and radiation. Rather, caregivers’ emotional challenges arose from the ways in which treatments contributed to an altered life for the caregiver, the overall effect of cancer on patient functioning and well-being, the demands of caregiving, as well as roles and responsibilities beyond caring for a person with CRC (e.g., related to family or employment). This represents a conceptualization of the cancer trajectory for caregivers that is not biomedically centered but rather reflects the day-to-day experiences of caregivers [28]. We also found that caregiver experiences of (5) navigating their relationships and (6) enduring unwanted change engendered emotions of consequence throughout the cancer trajectory. 

The adverse effects of caring for a patient with cancer on caregivers’ emotional well-being have certainly been acknowledged, with assessments focused on stress, mood, psychological distress, anxiety, and depression [29,30]. While these emotions were certainly evident in our study, these emotions alone failed to represent the range of emotions that characterized caregiving and that caregivers considered important. We found that additional emotions and feelings of worry, fear, powerlessness, uncertainty, frustration, emotional exhaustion, loneliness, anger, guilt, loss, and grief were experienced by caregivers. Considering the aim of developing and implementing CROs that are informed by robust conceptualizations of caregiver experiences, our research suggests that the reliance on CROs with a rather narrow focus on distress, anxiety, and depression is insufficient. Similar to PROs, the aim of CROs is to ensure that inferences, decisions, and actions in healthcare are informed by the caregiver’s point of view [31]. Thus, future research is urgently needed that examines the utility of CROs that measure a broader range of emotion-specific constructs to help mitigate the risk of not capturing the experiences or perspectives of various caregivers. This might also broaden to include experiences classified as feelings or moods. Where existing CROs do not yet exist, there is undoubtedly a need for CRO measurement development and validation among caregivers, and additionally among caregivers of patients with different cancer diagnoses. 

There is clearly variability in the emotional experiences of caregivers. A recent study by Decadt et al. [32] that examined CROs (distress and quality of life) among 1580 primary caregivers of patients with different cancer diagnoses in Belgium found the highest distress among caregivers who did not live with the patient and non-spousal caregivers. Our results provide insight into these findings such that both spousal and non-spousal caregivers described their desire to be always with the patient to quell their own worries about the patient but also out of recognition that they play a vital role in monitoring and ensuring patient well-being. The caregivers in our study expressed worry and distress when they were unable to be ever-present and provide the care they considered necessary, which was made harder when they did not live with the patient or they were unable to accompany the patient throughout treatment. However, we also found that caregivers who lived with the patient and perceived that they were the sole caregiver but who had multiple competing demands highlighted feeling alone, unsupported, depressed, overwhelmed, and emotionally exhausted. They also felt invisible and expendable when there was a lack of recognition for all they were doing. This aligns with prior evidence that caregivers’ actual or perceived personal networks significantly affect caregiver outcomes, such that not having an alternate caregiver for practical help increases caregiver burden, depression, distress, and anxiety [32,33,34]. Further, even perceived social support strengthens caregivers’ sense of coherence, which helps them to accept and act upon a cancer diagnosis in a caregiver role [35].

Considering the growing recognition of non-spousal caregivers of people with cancer and the influence of social support and networks on caregivers’ experiences, it is vital that cancer care professionals and systems shift their focus to a less individualistic and more relational notion of caregiving that goes beyond the caregiver-patient dyad. Future research is needed that considers CROs that assess these relational influences. Moreover, considering that most psychoeducational interventions target spouses, intervention development that targets non-spousal caregivers and that taps into social supports and networks could be promising [36].

While there has been a negligible shift in research and practice to considering the larger caregiver network, there is growing recognition of the interdependence of patient-caregiver dyads, with the caregiver usually considered the spouse [28,37,38]. Researchers have found that couples react to a cancer diagnosis as a unit or an emotional system, such that cancer can be thought of as a dyadic stress and stress management as dyadic coping [38,39]. Theories of dyadic coping suggest that both partners are mutually involved in the coping process, providing and receiving support, and engaging in joint problem-solving and shared emotion regulation, representing an interpersonal form of coping (regarded as a circular and bidirectional sequence) [38]. In their review, Traa and colleagues [38] found that coping styles characterized by open and constructive (cancer-related) communication, supportive behaviors, positive dyadic coping, and joint problem solving were related to higher relationship functioning, whereas dysfunctional communication patterns (e.g., protective buffering), unsupportive behaviors, and negative dyadic coping were related to lower relationship functioning. In our study, caregivers’ emotions of tension, conflict, anger, frustration, and guilt arose when there was a mismatch between the caregiver and patient in terms of desired/offered support, preferred coping styles, and expectations regarding relationship roles. These important emotions are perhaps related to difficulties or a breakdown in dyadic coping. Considering our findings, the context of theories of dyadic coping builds a compelling case, not only for future CRO research that includes tension, conflict, anger, frustration, and guilt, but also for the importance of considering the caregiver-dyad unit and dyadic coping. This area of study would further benefit from the exploration of patient-caregiver dyads that includes non-spousal caregivers. 

In oncology, the concepts of loss and grief appear to have been mainly investigated among caregivers in the context of a palliative diagnosis and/or following the death of the patient. Similar to other studies on family caregiving [40], our research highlights the importance of the loss and grief to caregivers of people with CRC, feelings that were present even from the time of diagnosis and not limited to before or after the patient’s end-of-life. Rather, study participants described loss and grief even when the patient had a curative diagnosis, which related to the major life disruption of cancer and recognition of their lives as forever changed. Though it is unclear from our research whether existing conceptualizations or theories of grief would be most relevant, it is worth drawing insight from the larger psychological literature. Grief, as a more general concept, encompasses an individual’s response to loss during any period of time, with symptoms of grief distinct from symptoms of anxiety and depression [41]. There is growing recognition of an intractable and disabling form of grief that is coupled with functional impairment and a failure to adjust to loss in the longer term. This is prolonged and/or complicated grief (now termed persistent complex bereavement-related disorder in the DSM-5, though conflict remains about whether prolonged grief disorder, complicated grief, and persistent complex bereavement disorder are distinct diagnostic entities) [42]. There is some evidence suggesting that any prolonged grief, negative caregiving experiences, and/or shifts in caregiver roles increase the likelihood of complicated grief. Thus, the development of CROs that identify common features of the grieving process as well as those associated with prolonged and/or complicated grief in the context of cancer could potentially be useful in providing psychosocial support that has both long- and short-term effects [43]. Such research is warranted. 

### Strengths and Limitations

The findings of this study must be considered in the context of study strengths and limitations. This qualitative Interpretive Description research represents the construction of evidence grounded in the accounts of caregivers and patients with CRC that is intended to be clinically useful, specifically in relation to CROs. Strengths of this research included the wide variation in participant demographics and experiences that enabled us to look for dissimilarity in our data as well as the inclusion of caregivers (spousal and non-spousal) and patients. The involvement of caregiver- and patient-partners; clinicians who represent nursing, medicine, social work, and counseling; and multidisciplinary researchers throughout the research facilitated robust discussion, which informed and enriched out study findings. Limitations of this research included our reliance on online recruitment methods that required participants to contact the study team. Thus, it is possible that our findings do not represent the full diversity of caregiver and patient experiences, particularly those who perhaps have lingering emotions or stresses that they would prefer not to discuss, including with a researcher they did not know. We only included caregivers of patients and patients themselves who received treatment, and thus, our findings are not transferable to situations where the patient did not receive treatment. We only conducted interviews in English with English-speaking participants, and so it is quite likely that we did not capture how the experiences of caregiving might differ across cultures. Moreover, while we attempted to recruit men who were caregivers, these individuals did not contact our research team as readily as women caregivers. Considering that interviews were conducted virtually, in the first 6 months of the COVID-19 pandemic, it is possible that participant recollections were reflective of and shaped by pandemic-related public-health measures and stresses, such as restrictions to in-person contact with family and friends or changes in family accompaniment or visitation during medical appointments. While our sample represented a wide range in participant age (caregivers 27 to 79 years; patients 31 to 84 years), there were only a couple of adults over 80 years of age. It is possible that the perspectives of older adults were under-represented because of our reliance on virtual recruitment and data collection. Similarly, the perspectives of structurally vulnerable individuals who have limited regular access to virtual means of communication are likely not represented in this study. Finally, our interpretation of the emotions of consequence excludes the positive aspects of caregiving that were present in our data but beyond the scope of this analysis. We intend to highlight these positive aspects in a future publication. 

## 5. Conclusions

Future efforts to develop, validate, and implement CROs into cancer care would benefit from robust evidence of the experiences and outcomes most important to caregivers, inclusive of the range of emotions highlighted in our research. While supportive care services are often available to caregivers as part of Canadian oncology services, the formal consideration of caregivers and the use of CROs as standards of care will require a substantial cultural shift in cancer care organizations, along with formal forms of support, resources, and processes of care. Yet, this shift holds tremendous potential to make real strides in actualizing the type of patient- and family-centered care that has tangible benefits for patients, caregivers, and the larger healthcare system.

## Figures and Tables

**Table 1:** Participant Demographic Characteristics.

	Caregiver (*n* = 25)	Patient (*n* = 37)
Characteristic	Number	Number
Mean Age (years)	55	65
Gender		
Woman	22	16
Man	2	21
Non-Binary	1	0
Relationship to the patient (You are the patient’s…)		
Husband/Man Partner	1	
Wife/Woman Partner	15	
Non-binary Partner	1	
Daughter	6	
Son	1	
Friend (Woman)	1	
Relationship to Caregiver (Your caregiver is your…)		
Husband/Man Partner		9
Wife/Woman Partner		17
Non-binary partner		1
Sister		1
Son		1
Cousin (Woman)		1
Friend (Woman)		1
Listed more than 1 caregiver role		6
Marital Status		
Married/Common-law/Living together	20	27
Divorced/Separated	1	4
Single	4	5
Widowed	0	1
Living Arrangement		
Living with the patient or their caregiver	16	29
Living alone	5	7
Other	4	1
Community Size		
Large city (100,000 or more residents)	14	20
Medium-size city (30,000 to 99,999 residents)	5	6
Small town/area (1,000 to 29,999 residents)	4	8
Small rural area (< 1,000)	2	3
Employment Status		
Full-time	8	6
Part-time	5	6
No	11	23
Other	1	2
Previously Cared for Someone with a Terminal Illness		
Yes	15	12
No	10	25
Cancer Stage of Patient		
1	3	2
2	1	10
3	6	16
4	8	4
Unknown	7	5
Patient Colostomy and/or Ileostomy		
Yes	11	18
No	14	19

**Table 2:** Overview of Caregiver Experiences Engendering Emotions of Consequence and Contributing Factors.

Caregiver Experiences	Emotions of Consequence and Feelings Expressed	Contributing Factors or Circumstances
**Caregiver experiences that engendered emotions of consequences during CRC diagnosis and treatment**		
Facing the patient’s life-changing diagnosis and an uncertain future	Shock Panic Fear Anxiety Distress Worry Powerlessness Uncertainty	Possibility of loved one dying Diagnosis unexpected Prior family history of cancer Patient kept symptoms hidden Hopes and dreams threatened Waiting for treatment plan Lack of confidence in caregiving Unfamiliar with impact of an ostomy on life Lack of caregiver-specific information
Needing to be with the patient throughout the “never-ending nightmare” of treatment	Fear Anxiety Distress Worry Unsupported	Possibility of loved one dying Unable to be with the patient Not living with the patient Patient had to travel Getting time off work Concern for patient all consuming Loss of reciprocal support Pre-existing mental health or traumatic cancer experiences
Bearing witness to patient suffering	Distress Sadness Frustration Depression	Patient experienced side effects Patient functioning declined Invasive medical interventions Patient suffering unnecessary and avoidable Visible pain and suffering Caregiver unfamiliar with caring role Sudden and unexpected role reversal
Being worn down by unrelenting caregiver responsibilities	Overwhelmed Emotional Exhaustion Lonely Unsupported Invisible Expendable	Persistent medical issues or complications Ongoing nature of caregiving (emotional and physical demands) Being the sole caregiver Multiple competing demands Getting time off work Lack of recognition or appreciation from others Reluctance to discuss challenges Unable to seek support Minimal opportunities for caregiver reprieve and emotional support
**Caregiver experiences that engendered emotions of consequence throughout the CRC trajectory**		
Navigating relationship	Tension Conflict Helpless	Changes in caregiver and patient roles and responsibilities Loss of reciprocal support Mismatch between caregiver and patient desire for support Patient resistance or acceptance of caregiver support Mismatch between caregiver and patient emotional coping
	Anger Frustration	Patient role trumped the patient relationship role Fear of upsetting or compromising patient well-being Focus on acceptance Lack of available caregiver support
	Guilt	The patient is ill while the caregiver is healthy Caregiver struggling when they think they need to be “strong” Taking time for self or engaging in self-care
Enduring unwanted change	Loss Grief	Future hopes and dreams Shifting priorities Change to identity and lifestyle Social disconnectedness and loss of friendship Loss of Intimacy, “intimacy on hold”

## Data Availability

The data that support the findings of this study are not available on request from the corresponding author nor are they publicly available because they contain information that could compromise the privacy of research participants.
